# Multivariate Dimensionality Reduction Approaches to Identify Gene-Gene and Gene-Environment Interactions Underlying Multiple Complex Traits

**DOI:** 10.1371/journal.pone.0108103

**Published:** 2014-09-26

**Authors:** Hai-Ming Xu, Xi-Wei Sun, Ting Qi, Wan-Yu Lin, Nianjun Liu, Xiang-Yang Lou

**Affiliations:** 1 Institute of Bioinformatics, College of Agriculture and Biotechnology, Zhejiang University, Hangzhou, P.R. China; 2 Research Center for Air Pollution and Health, Zhejiang University, Hangzhou, P.R. China; 3 Institute of Epidemiology and Preventive Medicine, College of Public Health, National Taiwan University, Taipei, Taiwan; 4 Department of Biostatistics, University of Alabama at Birmingham, Birmingham, Alabama, United States of America; Irvine, United States of America

## Abstract

The elusive but ubiquitous multifactor interactions represent a stumbling block that urgently needs to be removed in searching for determinants involved in human complex diseases. The dimensionality reduction approaches are a promising tool for this task. Many complex diseases exhibit composite syndromes required to be measured in a cluster of clinical traits with varying correlations and/or are inherently longitudinal in nature (changing over time and measured dynamically at multiple time points). A multivariate approach for detecting interactions is thus greatly needed on the purposes of handling a multifaceted phenotype and longitudinal data, as well as improving statistical power for multiple significance testing via a two-stage testing procedure that involves a multivariate analysis for grouped phenotypes followed by univariate analysis for the phenotypes in the significant group(s). In this article, we propose a multivariate extension of generalized multifactor dimensionality reduction (GMDR) based on multivariate generalized linear, multivariate quasi-likelihood and generalized estimating equations models. Simulations and real data analysis for the cohort from the Study of Addiction: Genetics and Environment are performed to investigate the properties and performance of the proposed method, as compared with the univariate method. The results suggest that the proposed multivariate GMDR substantially boosts statistical power.

## Introduction

Many complex disorders such as asthma, diabetes, cardiovascular disease, Alzheimer’s disease, hypertension, mental disorders, and drug addictions are generally multifaceted phenotypes, measured by a set of scales and/or intermediate phenotypes that can be correlated as the outputs of a metabolic network with many interwoven pathways [1]–[4]. Longitudinal data at multiple time points may also be gathered for investigating the trajectory of a phenotype [5]. Furthermore, two-stage multiple testing analysis, i.e., multivariate analysis for grouped phenotypes followed by univariate analysis for the phenotypes in the significant group(s), offers a plausible way to increase statistical power because the traditional Bonferroni correction is usually overly stringent [6], [7]. Thus, a multivariate approach is highly demanded for tracking down pleiotropic contributors to complex multifaceted disorders and genetic effects underlying dynamic traits, and for increasing test power [8]–[12]. In addition, the multivariate strategy can also provide an attractive framework for data integration of several datasets of multi-omics features.

On the other hand, as a natural property of complex networks and the widespread intermolecular dependence in gene regulation and biochemical and metabolic systems, the presence of interactions appears to be the norm rather than the exception. Ever-growing evidence has pointed to the view that risk factors act in concert, rather than isolated, to affect complex diseases–the joint actions or interactions of multiple genetic and non-genetic factors play an important role in the etiology of complex diseases [13]–[16]. The elusive but ubiquitous interactions present one of greatest challenges for genetic epidemiologists because they make the risk factors underlying complex phenotypes elude traditional single factor-based hunting strategies [14], [16], [17]. A large amount of research has been devoted to the development of new analytical approaches for the detection and investigation of those interactive determinants involved in complex diseases [18].

The data reduction approaches such as the multifactor dimensionality reduction method (MDR) [19]–[21], the combinatorial partitioning method (CPM) [22], and the restricted partition method (RPM) [23], represent a promising tool for a better identification of simultaneous associations and interactions among multiple risk factors. To circumvent the weaknesses of existing combinatorial approaches [24], we developed a generalized MDR (GMDR) statistical framework that is applicable to diverse phenotypes in population-based and family-based studies and allows adjustment for covariates [25], [26]. To date, however, most of the new methods in the literature are univariate, only offering to run separate analysis on one trait at a time. Such a separate analytical strategy does not utilize the information contained in multivariate data. In this article, we propose generalized linear model-, quasi-likelihood model- and generalized estimating equations model-based multivariate combinatorial approaches to detecting gene-gene and gene-environment interactions underlying multiple complex traits. We conduct a series of computer simulations to demonstrate the powerfulness of the proposed methodology. Finally, the proposed methodology is illustrated by an application to a set of real data on nicotine dependence (ND) in the cohort from the Study of Addiction: Genetics and Environment (SAGE).

## Materials and Methods

### Ethics Statement

The datasets used for the analyses described in this article were obtained from the database of Genotypes and Phenotypes (dbGaP). All the study protocol and forms/procedures were approved by the Institutional Review Board of the University of Alabama at Birmingham.

In the original MDR method for a case-control study [19], a set of *m* attributes either genes or discrete environmental factors are chose to span an *m*-dimensional contingency table. Each subject is allocated into a cell in this *m*-dimensional space based on the observations on these attributes and every nonempty cell can be labeled as either “high-risk” if the ratio of cases to controls in the cell is larger than a pre-specified threshold or “low-risk” otherwise. A new dichotomous attribute (i.e., a classification model) is formed by pooling the high-risk cells and the low-risk cells into the high-risk group and the low-risk group, respectively, thus changing the space of the data from originally higher dimensions to one dimension. The resulting model is evaluated in its ability to classify the phenotype; accuracy, defined as the proportion of the correct classifications (i.e., cases in the high-risk group and controls in the low-risk group), is a commonly used measure. Cross-validation and/or permutation testing can be integrated into the above process for evaluation of model, and the optimal subset(s) of features can be selected in terms of the classification ability measured by accuracy or its derivatives such as *p*-value.

While sharing the same variable construction algorithm as in MDR, GMDR uses a general statistic, instead of affection status, to classify the two divergent groups. The statistic of an individual corresponding to a certain cell in a given contingency table can be generally expressed as the product of its membership coefficient belonging to this cell and its residual under the null hypothesis, which will be elaborated in the following subsections, respectively.

### 2.1. Statistical models and residuals under the null model

Multivariate traits can be represented by an appropriate statistical model corresponding to data nature; generalized linear model (GLM) [27], quasi-likelihood model (QLM) [28] and generalized estimating equations (GEE) model [29] are the commonly used models. All the GLM, QLM and GEE model have the same form of linear predictor and link function. Considering a. -dimensional response vector 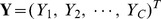, denote the expectation of 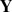 by 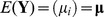. Assume that a set of explanatory variables influence the outcomes and there is an invertible link function 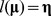 relating the mean to the linear predictor, which can be expressed as,
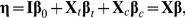
(1)where 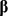 is the effect vector probably consisting of 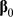, 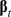 and 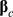 for the intercept(s), the target effects of interest (i.e. gene-gene and/or gene-environment interactions), and the covariate effects, respectively, 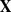 is the corresponding design matrix consisting of block matrices 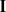, 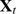 and 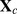, and 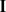 is an identity matrix.

The linear predictor can be used for various scenarios. For a repeated measurement study, 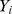 may have the same parameters 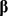 and regressor values 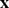, and thus the design matrix 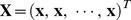. In a clustered design or a longitudinal study, the components of 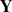 may share the same 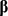, but have their own regressors 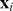
s, and thus 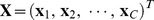. For grouped phenotypes in a two-stage multiple testing, each 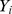 has the component-specific predictor values and parameters including 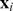 and 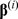, and thus resulting in the block effect vector and block incidence matrix, respectively, as follows,
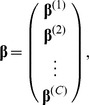
and
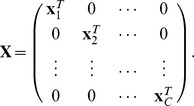



In application to detection of overall effects and/or pleiotropic effects on multiple traits, the components of 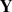 may share the same regressor 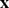, but have component-specific parameters 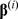.

The residuals can be computed under the null hypothesis of no target effects, i.e., 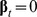, and the estimation methods for different models are summarized as follows.

#### 2.1.1. Multivariate generalized linear model

A GLM is characterized by three parts: the response distribution, the linear predictor, and the link function between the linear predictor and the mean of the response variable. Specifically, the density function of a GLM has such a general form,
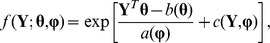
where 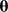 is the location parameter vector, 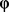 is the dispersion or scale parameter vector, 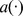, 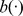, and 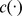 are known functions to specify a member of the exponential family. The expectation and variance of 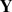 are, respectively,



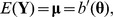
and
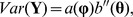
where 
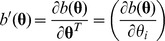
 and 
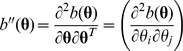
 are the first-order and second-order derivatives of 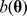, respectively. Denote 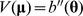, called variance function, to highlight that 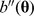 depends on 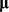. The link function 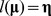 relates the mean to the linear predictor in Equation (1).

The score of likelihood for a set of independent observations 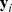 (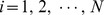) is,
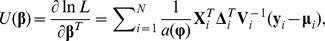
where 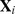
’s are the design matrices of observation *i*, 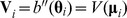, and 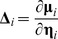.

The GLM can be fitted by maximum likelihood (ML) estimation method. Note that estimation of 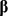 does not require knowledge of 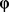, implying that one may first estimate 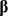 and then estimate 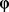 based on the estimate 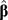. The ML estimator can be derived by setting the score equal to zero and solving the resulting estimating equations. The Newton-Raphson method and the Fisher's scoring method are two well known methods implemented with iteratively weighted (or reweighted) least squares method (IWLS) for finding ML estimates in GLMs when there is no closed form of ML estimates available.

The unknown scale parameter 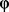 can be estimated separately from 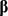 after computing residuals using 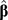. Although this parameter can, in principle, be estimated by maximum likelihood as well, it is more common to use a “method of moments” estimator. Unbiased estimator of 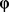 can be obtained via Pearson’s Chi-square statistic as,
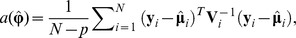
where 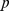 is the number of independent parameters estimated in 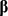 (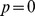 if 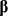 is known).

Under the null hypothesis of no target effects (i.e., 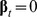), we fit 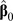 and 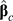 as well as 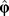 to data. Then, the residuals can be computed by,
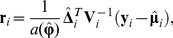
(2)where 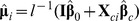 and 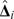 is an estimate of 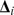 in which 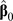, 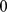 and 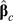 are used in place of 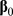, 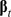 and 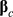, respectively.

#### 2.1.2. Multivariate quasi-likelihood model

QLM can be used when only partial information on the data features is available: how the mean is related to the explanatory variables and how the variance of an observation is related to its mean. Compared to GLM, QLM only specifies the link function and the relationship between the first two moments but does not necessarily specify the complete distribution of the response variable. In QLM, quasi-likelihood function is constructed to mimic a proper likelihood function. A quasi-likelihood has the same properties as log-likelihood and the quasi-score function can be formulated by differentiating the quasi-likelihood function. The quasi-score behaves like the score in GLMs. Quasi-likelihood models can be fitted using a straightforward extension of the algorithms used to fit GLMs. The estimating equation for the residuals under the null hypothesis is the same as Equation (2).

#### 2.1.3. Generalized estimating equations model

GEE model is an extension of GLM and QLM [29]–[31]. GEE model requires only specifying a functional form for relationships between the outcome variable and the explanatory factors and between the mean and the variance of the marginal distribution, avoiding the need to model the multivariate distribution and covariance structure for data. Specifically, letting 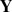 be a group of response variables, suppose that (1) there is a link function relating the expectation of 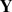 to a linear predictor in Equation (1), 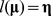; and (2) the variance is a function of the mean, 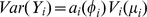, 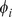 is the scale parameter, and 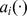 and 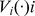 are some known functions.

Considering a set of data 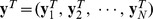 that is decomposed into 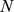 strata and the 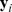
’s are uncorrelated with each other, the estimating equations are formed via a set of 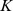 score or quasi-score equations,
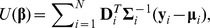
Where 
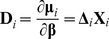
, 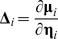, 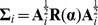 is a working variance with a given correlation structure, 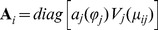, a diagonal matrix with 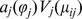 as the 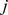 th diagonal element, and 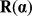 is a working correlation matrix that may depend on some unknown parameter vector 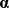. 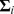 does not need to be equal to the true covariance matrix, although the closer it is to the true one, the better precision the estimates will achieve.

The estimates of 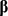 can be found by solving 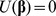. Estimation is typically accomplished through a series of iterations between a modified Fisher’s scoring algorithm for 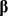 and moment estimation of correlation parameters 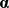 and scale parameter 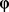. Given current estimates 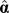 and 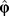 of the nuisance parameters, the following modified iterative procedure is for 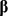,
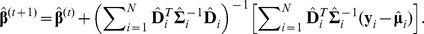



The working correlation matrix 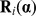 and 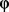 are estimated by the method of moments. Using the current values of parameters calculates the current Pearson residuals defined as,
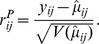



Then, 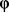 can be estimated by,
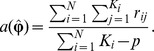



The specific estimator for 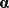 depends on the choice of 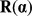; the general approach is by the function of,
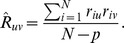



After fitting the model under the null hypothesis, the residuals can be computed for several different purposes, e.g., a repeated measurement study, a longitudinal study, a clustered design, and multivariate analysis,
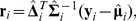
(3)where 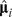, 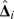 and 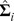 are, respectively, the GEE estimates of the mean, matrices 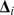 and 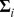 under the null hypothesis, 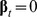. When all the components of 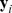 have the same target effect parameter 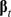 as in a repeated measurement study, the residuals can be further averaged over these measurements for a better estimation,


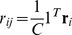
, 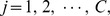 (4)where 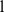 is a vector of which all components are 1 and 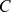 is the dimensionality of 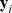.

### 2.2. Membership coefficient

Membership coefficients that form matrix 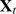 in Equation (1) are used to characterize to which cell(s) a subject can be allocated in the space spanned by a set of target factors. To simplify our presentation, we consider here the samples coming from a homogeneous population. For other cases such as samples from an admixed population and family samples, please refer to our reports elsewhere. The membership coefficient of an unrelated subject is defined as an indicator variable,
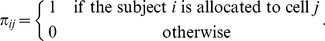
(5)


### 2.3. Multifactor Dimensionality Reduction Algorithm

The statistic can be defined for various scenarios. In the case where the components of 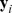 have the same target effects 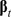 whether or not the predictor vectors of the components are distinct, the statistic of component 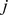 in observation 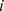 with respect to cell 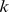 in a given contingency table can be computed by (treating as individual 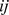 and cell 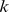),
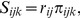
(6)where 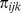 is the membership coefficient denoting component 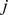 in observation 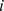 pertaining to cell 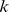 in a given contingency table; all 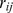 s of observation 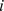 (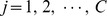) are the same in a repeated measurement study; and all 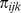 s of observation 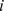 (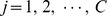) for cell 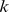 are the same in a repeated measurement study and probably in a longitudinal study. In application to detecting the overall effects and/or pleiotropic effects of determinants on a group of multivariate phenotypes, the statistic is considered as (treating as individual 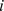 and cell 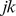),
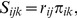
(7)where 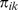 is the membership coefficient denoting individual 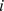 belonging to cell 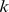 in a given contingency table. In detection of the pleiotropic effects, the aggregation is also suggested to use for each individual [32],



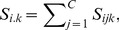
(8)For convenience of notation, we use 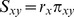 hereinafter to denote the statistic of individual 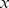 pertaining to cell 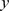 by treating 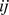 as a new individual 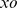 and cell 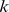 as 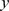 in (6), and treating cell 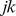 as a new cell 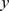 and individual 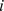 as 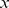 in (7).

The statistic defined above reflects a putative association between the phenotype(s) of interest and the target factor(s), offering a possibility for variable construction to create plausible new attributes that maximize the residual phenotypic correlation. When the null hypothesis holds true, the association is expected to be zero and the classification model is formed purely by chance. The variable construction process is illustrated with the 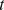-fold cross-validation procedure although such a cross-validation is not always necessary as other techniques such as permutation testing may determine whether a classification model is beyond chance. The MDR process is described briefly as follows.

In Step 1 the data are randomly split into 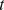 equal or nearly equal parts for 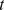-fold cross-validation. One subset is used as the testing set and the remained as the independent training set. Then, Steps 2 through 5 are run for the training set to construct a new dichotomous attribute and Step 6 for the testing set to evaluate the fitness of the new attribute(s). In Step 2, a subset of 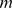 factors is selected from all 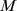 genetic and/or discrete environmental factors, giving rise to a total of 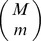 distinct subsets. In Step 3, each such subset corresponds to a 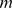-way contingency table and each membership component of a subject does to one cell in the table. The statistic value of each nonempty cell can be averaged over by 
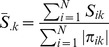
 for cell 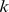. Each nonempty cell is labeled either high-valued if its average statistic value is not less than some threshold 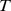 (
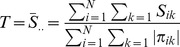
 by default), or low-valued otherwise. In Step 4, a new attribute is created by pooling high-valued and low-valued cells into two contrasting (i.e., high-valued and low-valued) groups, representing that best captures the correlation between this set of classification factors and the phenotype(s). In Step 5, the classification accuracy, defined below, can be assessed for each contingency table. The best model(s) can be identified among all the possible 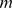-way contingency tables based on classification accuracy. In Step 6, the independent testing set is used to evaluate the testing accuracy for the best model among those with different dimensionalities identified in Step 5. If the classification model is formed purely by chance, it will give a null testing accuracy of 0.5. The significance test can be implemented based on permutation testing, nonparametric sign testing and asymptotical normal test for testing accuracy.

The accuracy is defined by,
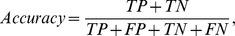
where TP is True Positive, defined as having a high value in the high-valued group, TN is True Negative, defined as having a low value in the low-valued group, FP is False Positive defined as having a low value in the high-valued group, and FN is False Negative defined as having a high value in the low-valued group. The balanced accuracy [33] is also used,



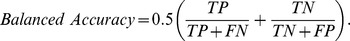



The statistic is by nature quantitative. However, the above accuracy, measured by the proportion of correct classifications in all classifications, only makes use of the qualitative information in the statistics, i.e., whether or not the statistic value of an individual in the high-valued (low-valued) group is larger (less) than the threshold. As an alternative, the accuracy may also quantitatively be measured by the differences in the averaged statistic values between the high-valued and the low-valued groups [26].

### 2.4. Simulation Study

To verify the validity and demonstrate the high statistical power of the new approach, we performed extensive simulations for continuous phenotypes. To simplify our exposition, here we considered a total of 10 unlinked loci with equi-frequent causative alleles, including two, three, and four disease-causing genes. Hardy-Weinberg equilibrium and linkage equilibrium were assumed in the simulations. Genotype data were simulated for 1,000 individuals with minor allele frequency of 0.3. Bivariate traits were simulated under the digenic espistatic (i.e. two functional loci involved), trigenic (i.e. three functional loci involved), and tetragenic (i.e. four functional loci involved) interaction models commonly used in simulation studies, for example antidiagonal model (i.e., genotypes *AAbb*, *AaBb* and *aaBB* were considered as high-value groups and the rest as a low-value group), 3 uppercase-letter model (3ULM) (i.e., genotypes with three uppercase were set as a high-valued groups and the rest as a low-valued group), 4 uppercase-letter model (4ULM) (i.e., genotypes with four uppercase letters were set as high-value groups and the rest as a low-value group), in which the marginal effects of each disease locus were close to zero. For each individual, two continuous traits were simulated conditional on genotype data, interaction model, heritability of each trait and correlation between traits. Phenotypes were generated based on the following multivariate linear model,
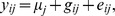
where 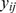 denotes phenotype 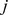 of individual 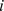, 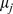 is the population mean of phenotype 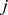, 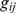 is the joint effects of surveyed genes (or target environmental factor when GE interactions are involved) in subject *i* on phenotype *j*, and 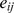 is the residual effects with distribution 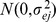.

Genetic effect 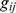 is defined as follows,
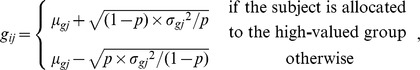
where 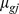 is the mean genetic effect of the phenotype 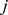, 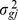 is genetic variance of joint effects for the phenotype 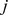, 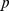 is the multilocus genotype frequency of the high-valued group.

In the simulations, the multiple functional loci affected the two simulated traits simultaneously. Thus we could generate the phenotypic values of the two simulated traits of an individual *i* via a bivariate normal distribution with the mean 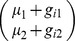 and the variance-covariance matrix 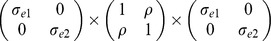, where 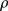 is the residual correlation coefficient. In the present simulations, the targeted gene-gene interaction controlled each trait equally (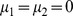, 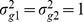, 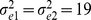), responsible for 5% (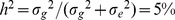) of the total phenotype variance for each trait. The residual effects were responsible for the remaining 95% of the phenotypic variance and different residual correlations (i.e., 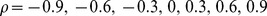) were considered in the simulations. Other shared factors were not considered here.

The statistics of all individuals for GEE-based GMDR (GEE-GMDR) were computed based on equation (8) using R software package geepack [34]. Then the GEE score statistics were input into the GMDR software to identify the best interaction model. For the purpose of comparisons, separate analysis of phenotypes was also performed using the original GMDR method. A threshold of 0 was used to determine whether a cell is high-valued or low-valued in both the methods in the subsequent analysis. We conducted an exhaustive search with 10-fold cross-validation for all possible one- to nine-locus models in our simulations. The average cross-validation consistency (CVC) and test accuracy (TA) as well as their standard error means (SEMs) were summarized based on 200 simulation replicates. Additionally, Type I error rate and statistical power were also calculated on the basis of the null TA distribution for each scenario under GEE-GMDR and GMDR, respectively. The thresholds for TA at 5% and 1% significance levels were determined through an empirical distribution of TA constructed from permutation testing with 1,000 replicates. We randomly permuted the score statistics to generate pseudo samples for its null distribution in which the potential association between the multilocus genotype and the phenotypes of interest. And then the cross-validation GMDR was performed with the permuted samples, namely, the training data were used to identify the model and the testing data were used to evaluate the model. As the testing samples are independent of the training samples in which the model selection is involved, the TA will randomly fluctuate around 0.5 under the null of no target effects. The TAs from the permutations formed an empirical null distribution. Power and Type I error rate were calculated based on the proportion of the significant models detected in 200 and 1,000 simulations, respectively, with its TA values no less than the cutoff point for each scenario across seven levels of the residual correlation.

### 2.5. A Case Study on Nicotine Dependence

To illustrate use of the GEE-GMDR approach proposed here, a real data set from the Study of Addiction: Genetics and Environment (SAGE) was analyzed to identify interactions among genes. A large proportion of SAGE samples were unrelated except a few siblings. After quality control, a total of 3,897 individuals from three subsamples: the Collaborative Study on the Genetics of Alcoholism (COGA) (1,178 individuals), the Collaborative Study on the Genetics of Nicotine Dependence (COGEND) (1,427 individuals) and the Family Study of Cocaine Dependence (FSCD) (1,292 individuals) were obtained. Using Illumina Human 1M platform, 1,069,796 SNP markers were genotyped for each participant. Self-reported ethnicities indicate that about 35% of the participants are black and 65% are white. Detailed genotype information and demographic characteristics of SAGE cohort can be obtained from the database of Genotypes and Phenotypes (dbGaP) through dbGaP accession number phs000092.v1.p. Three common different measurements of ND were selected from the recorded traits: the lifetime score on FagerstrÖm Test for Nicotine Dependence (FTND), the DSM4 Nicotine Dependence (DSM4ND) and the largest number of cigarettes smoked in 24 hours (MC).

We excluded SNPs that had missing genotype rate >0.1, minor allele frequency <0.05 and a Hardy-Weinberg equilibrium test *p*<10^−7^ using PLINK software [35]. In total, 744,511 SNP markers were left after quality control. A total of 2,082 individuals were available for the phenotypic traits and also passed the quality control. We also generated a pruned subset of SNPs that are in approximate linkage equilibrium with each other using PLINK software. With the SNP information (dbSNP, Build 135) and the remained SNPs passing the control process, 5 SNP markers in the nicotinic acetylcholine receptor (nAChR) *α4* subunit (*CHRNA4*), 3 in the nAChR *β2* subunit (*CHRNB2*), 56 in the neurotropic tyrosine kinase receptor 2 (*NTRK2*, also known as the tyrosine kinase receptor gene, *TrkB*), and 18 in the brain derived neurotropic factor (*BDNF*) were chosen to detect gene-gene interactions among the four genes. In total, 15,120 (5×3×56×18) tetragenic interactions with one SNP from each of the four genes were examined.

Owing to the fact that self-identified ethnicity often partially reflects one’s genetic ancestral origins, especially for populations that have complicated migration or admixture histories, the principal components analysis was performed using GCTA software for the SAGE data in which both unrelated samples and relatives are included to identify the population structure [36]. The residual score statistics of GEE-GMDR were computed using methods described in the above subsection with gender and the top five principal components as covariates. Permutation testing was conducted to obtain empirical distribution of test accuracy based on 1,000 shuffles. According to the central limit theory, the *p*-value can be approximately calculated under the null distribution by the approximated Z score, which is 
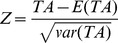
. Due to the computational burden for permutations, only the tetragenic model, passing the sign test for test accuracy implemented in the GMDR software at the significant level of 0.05, would be evaluated using permutation testing. For the purpose of comparison, we also used GMDR to analyze the three traits individually.

## Results

### Computer Simulations

**Figure 1:**
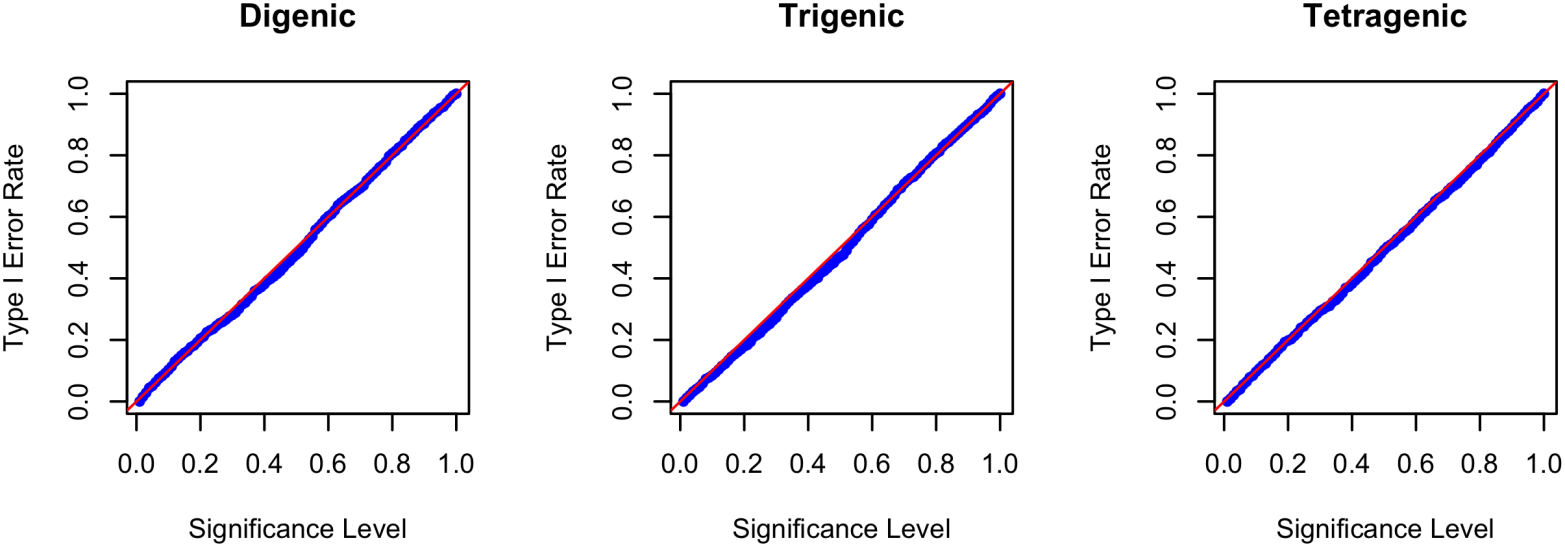
Quantile-quantile plot of significance level and Type I error rate. The Type I error is evaluated by GEE-GMDR method with digenic, trigenic and tetragenic models in presence of no gene-gene interaction and no residual correlation. The reference line is a diagonal line with unit slope through the origin. An unbiased method is expected to give the points falling on or near the reference line (i.e., Type I error rate is very close to the nominal level).

The Type I error rates under no residual correlation are presented in Table 1 for the digenic, trigenic and tetragenic interactions of GEE-GMDR and GMDR approach. All the estimates of Type I error were close to the nominal levels. The Type I error rates at the 0.05 significance level were 0.040, 0.054 and 0.048 with the digenic, trigenic and tetragenic interactions for GEE-GMDR method. The Q-Q plot of significance level vs. Type I error rate was well consistent with the theoretical expectation for GEE-GMDR method (Figure 1). The results with different residual correlations were similar (data not shown). The simulation results suggested that the new algorithm had correct Type I error rates, supporting the validity of the proposed procedure.

**Table 1:** Type I error rates for GEE-GMDR and GMDR methods.

Method^a^	GEE-GMDR			GMDR-T1			GMDR-T2		
Significance level	Digenic	Trigenic	Tetragenic	Digenic	Trigenic	Tetragenic	Digenic	Trigenic	Tetragenic
0.05	0.040	0.054	0.048	0.052	0.053	0.050	0.050	0.056	0.047
0.01	0.006	0.008	0.011	0.014	0.010	0.010	0.006	0.014	0.007

a GEE-GMDR is the GEE-GMDR analysis for the simulated bivariate traits, GMDR-T1 is the univariate GMDR analysis for trait 1, and GMDR-T2 is the univariate GMDR analysis for trait 2.

**Figure 2:**
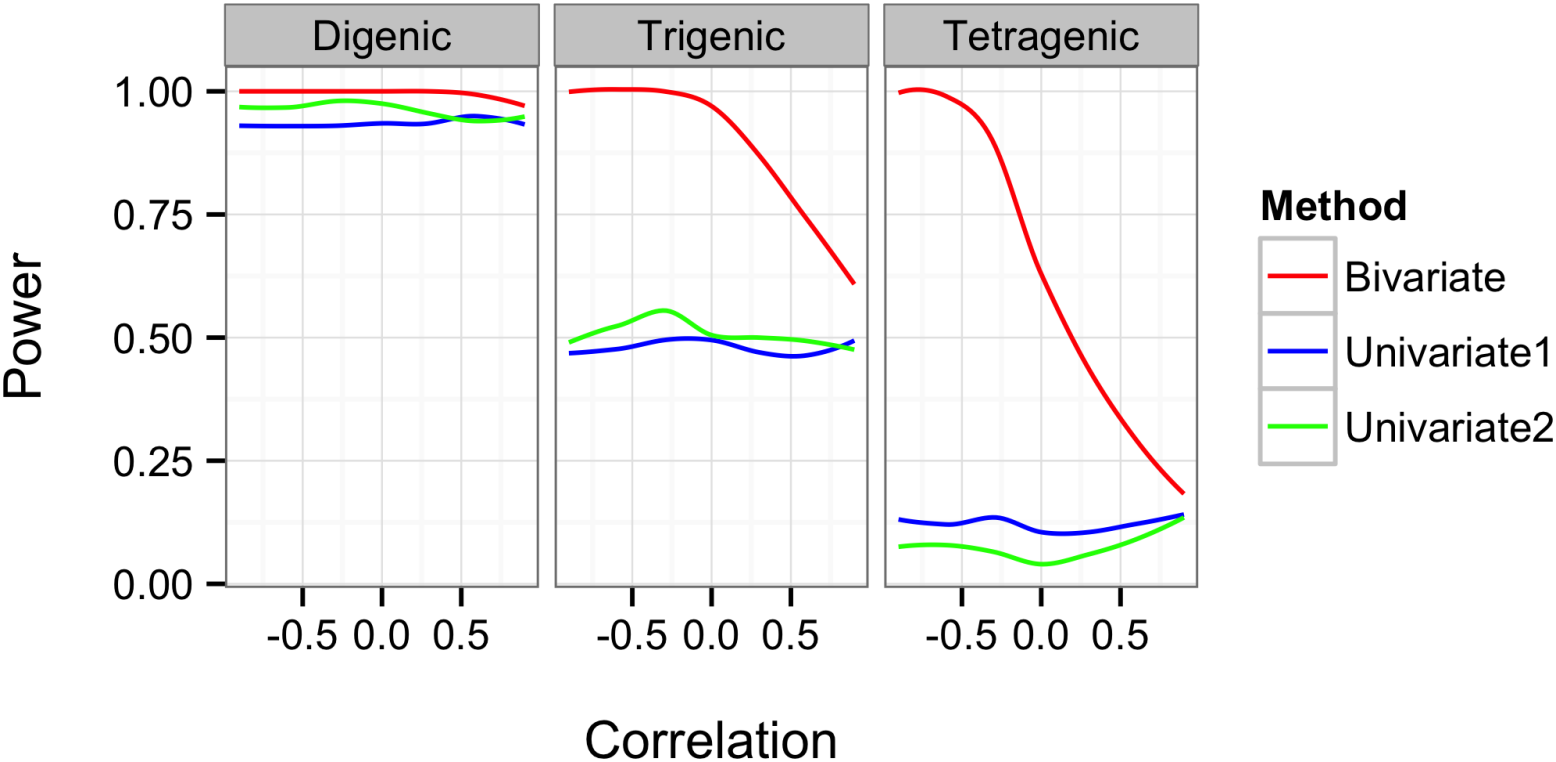
Comparison of statistical power between univarate GMDR method and GEE-GMDR under digenic, trigenic and tetragenic interaction models. The horizontal axis represents different residual correlations. The empirical statistical power is defined as the proportion of significant true models at 5% level in 200 simulations.

Table 2 displays the means and SEMs of both test accuracy and cross-validation consistency for two simulated continuous traits under no residual correlation. As expected, both bivariate analysis of GEE-GMDR and univariate analysis of GMDR always reach maximum test accuracy and cross-validation consistencies at the particular multilocus models with the same gene number as the simulated models, suggesting that the models with two, three and four functional genes could be correctly identified. Compared with the univariate analysis of GMDR, the GEE-GMDR bivariate analysis had higher test accuracy and cross validation consistency under the correct analytical model, for example, the means of test accuracy and cross validation consistency with GEE-GMDR are 0.665 and 10.000 for digenic model, 0.630 and 9.940 for trigenic model, 0.587 and 8.580 for tetragenic model, respectively, whereas those with GMDR for trait 1 (trait 2) are 0.617 (0.618) and 9.940 (9.975) for digenic model, 0.573 (0.572) and 8.655 (8.585) for trigenic model, 0.528 (0.526) and 5.725 (5.715) for tetragenic model. The results show that the GEE-GMDR method can utilize more information of multiple phenotypes and effectively improve test accuracy and cross-validation consistency. The test accuracy, cross validation consistency, and power (Fig. 2) seemed to be decreased for these three interactions patterns (i.e., digenic, trigenic, tetragenic) and this may be partly due to a lower frequency of the high value group and inflation of sampling error with increasing multilocus genotype given a limited sample size.

**Table 2:** Comparison of Cross-Validation Consistency (CVC) and Test Accuracy (TA) between GEE-GMDR and the GMDR method for two simulated continuous traits.

	GEE-GMDR^d^ (Mean±SEM)		GMDR-T1^e^ (Mean±SEM)		GMDR-T2^f^ (Mean±SEM)	
Model and No. ofloci	CVC	TA	CVC	TA	CVC	TA
Digenic^a^:						
1	9.010±1.446	.573±.025	8.445±1.886	.546±.035	8.500±1.616	.546±.027
***2***	***10.000±.000***	**.** ***665±.019***	***9.940±.433***	**.** ***617±.023***	***9.975±.211***	**.** ***618±.021***
3	6.680±2.056	.647±.024	6.790±2.147	.597±.032	6.460±2.025	.594±.029
4	5.550±2.126	.624±.029	5.485±2.141	.575±.037	5.435±2.071	.576±.031
5	5.230±2.131	.603±.033	5.105±1.911	.557±.035	4.820±2.066	.554±.032
6	5.050±2.017	.583±.033	4.695±1.844	.543±.033	4.765±1.915	.542±.034
7	4.970±1.977	.562±.035	4.825±2.165	.530±.037	5.195±1.999	.535±.034
8	5.340±2.036	.550±.040	5.465±2.140	.528±.044	5.250±2.034	.526±.038
9	6.705±2.110	.545±.050	6.500±2.088	.524±.050	6.720±2.178	.533±.052
Trigenic^b^:						
1	8.625±1.700	.551±.029	8.095±2.017	.533±.034	7.720±2.020	.527±.031
2	7.805±2.017	.568±.031	6.655±2.142	.536±.034	6.535±2.117	.530±.034
***3***	***9.940±.409***	**.** ***630±.023***	***8.655±2.031***	**.** ***573±.038***	***8.585±2.031***	**.** ***572±.035***
4	6.765±2.069	.607±.027	6.320±2.250	.559±.037	6.090±2.110	.556±.033
5	5.685±1.996	.582±.030	5.210±1.989	.541±.035	5.020±2.096	.540±.034
6	5.375±2.075	.565±.033	4.720±2.008	.529±.035	4.805±1.922	.527±.033
7	5.090±2.048	.544±.035	4.585±2.016	.520±.035	4.940±2.041	.522±.034
8	5.395±2.020	.534±.041	5.460±2.083	.521±.041	5.335±2.169	.519±.041
9	6.770±2.066	.535±.052	6.545±2.147	.518±.052	6.740±2.247	.526±.050
Tetragenic^c^:						
1	8.060±1.764	.537±.029	7.730±2.076	.524±.036	7.465±2.025	.537±.029
2	7.000±2.271	.546±.034	6.415±2.284	.526±.036	5.870±2.251	.518±.035
3	6.330±2.291	.548±.037	5.285±2.302	.522±.035	5.245±2.135	.519±.033
***4***	***8.580±2.132***	**.** ***587±.037***	***5.725±2.420***	**.** ***528±.041***	***5.715±2.213***	**.** ***526±.034***
5	6.105±2.125	.564±.037	4.865±2.126	.524±.036	4.980±1.985	.521±.030
6	5.190±2.068	.543±.038	4.675±1.995	.519±.033	4.605±1.967	.517±.035
7	4.860±1.881	.530±.036	4.830±1.881	.516±.032	4.840±2.087	.515±.035
8	5.280±2.101	.525±.042	5.350±2.109	.514±.041	5.195±2.034	.511±.038
9	6.845±2.030	.527±.050	6.470±2.126	.515±.051	6.725±2.203	.521±.050

a The genotypes with two uppercase-letter alleles (i.e., AAbb, AaBb, aaBB) are set as high-risk group and the rest as the low-risk group.

b The genotypes with three uppercase-letter alleles are set as high-risk group and the rest as the low-risk group.

c The genotypes with four uppercase-letter alleles are set as high-risk group and the rest as the low-risk group.

d GEE-GMDR is the GEE-GMDR analysis for the simulated bivariate traits.

e GMDR-T1 is the unvariate GMDR analysis for trait 1.

f GMDR-T2 is the univariate GMDR analysis for trait 2.

Figure 2 presents the comparison of the power of the two different analysis approaches to detect gene-gene interactions with pleiotropic effects based on a variety of residual correlation coefficients under digenic, trigenic, and tetragenic models. It indicated that the increase in power for multivariate analysis of GEE-GMDR, relative to univariate analysis of GMDR, depended on the residual correlation for trigenic, tetragenic models and the improvement is dramatic in some situations. GEE-GMDR performs particularly better for negative residual correlation coefficients. This observation is consistent with the previous results of multivariate research [6], [37]–[39]. However, the trend is not so apparent for digenic model. It may mostly be due to the extremely high power of digenic model (both approximate 100%) under our simulation set and the difference in power between two analytical methods cannot be distinguished. In some simulated cases with reduced allele frequency or genetic variance, however, we observed the same trend of power decrease with increase in the residual correlation (data not shown).

In summary, GEE-GMDR method maintains appropriate Type I error rate and thus is a valid test. The comparison of the multivariate analysis of GEE-GMDR with the univariate analysis of GMDR to identify combinations of multiple target genes demonstrates that the GEE-GMDR has higher or at least equal power in most situations. The GEE-GMDR method is able to substantially improve test accuracy, cross-validation consistency and power with inclusion of covariance structure of multiple phenotypes in GEE model.

### Application to Nicotine Dependence Data

**Figure 3:**
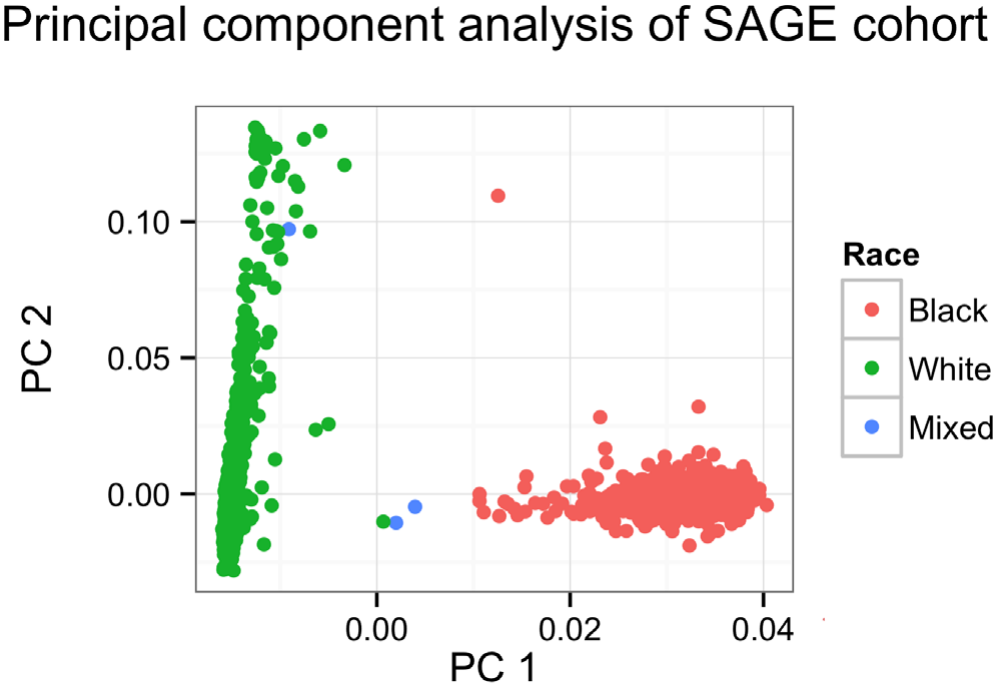
The principal components analysis for SAGE. The first two principal components are plotted to represent genetic background of the SAGE.

Figure 3 from the principal components analysis shows that the SAGE cohort could be clearly separated into three groups that are roughly consistent with self-reported ethnicity (black, white, and admixed). Although the race and ethnicity are thought to reflect unobserved environmental factors such as diet and family information, the self-reported race and ethnicity are not clearly defined and may not reflect the underlying complexity of them. Thus, it is more appropriate to estimate genetic background from genotype data, such as PCA, and use such information in the analysis [40]. The average correlations among the three phenotypes are 0.52 and they are highly correlated with each other.

**Figure 4:**
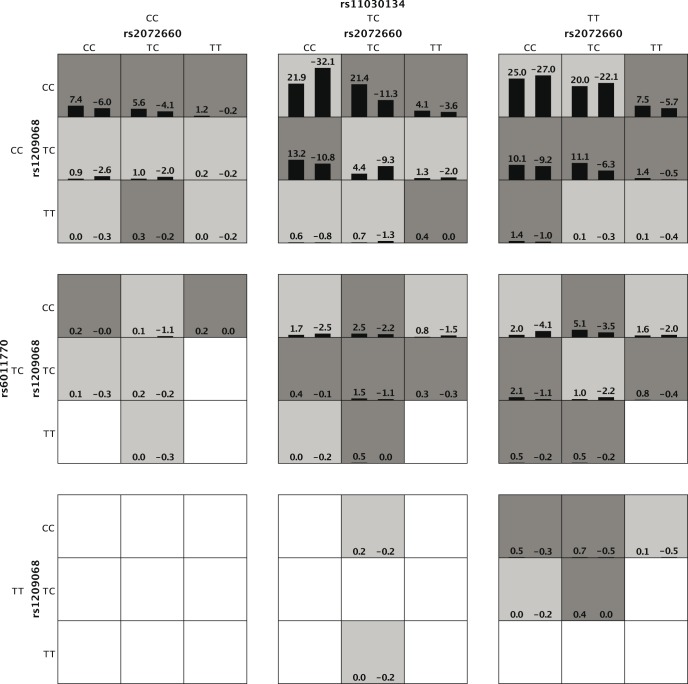
The interaction pattern among rs2072660-rs1209068-rs11030134-rs6011770. The left bar in each nonempty cell denotes a positive score and the right bar a negative score. High-risk cells are indicated by dark shading, low-risk cells by light shading, and empty cells by no shading. Note that the patterns of high-risk and low-risk cells differ across each of the different multilocus dimensions, presenting evidence of epistasis.

Using our new multivariate method and univariate method, 15,120 tetragenic interaction models were examined with one SNP from each gene. The normal distribution of the test accuracy was consistent with our expectation (data not shown). The GEE-GMDR method detected the most significant tetragenic interaction model (rs2072660- rs1209068- rs11030134- rs6011770) with an empirical *p* value of 2.62e-04 and test accuracy of 0.5780, supporting our hypothesis of a possible interaction among *CHRNB2*, *NTRK2*, *BDNF*, and *CHRNA4* underlying ND (Table 3). Detailed information on the SNP of the most significant tetragenic interaction model was given in Table 4. The *p* value of the identified model using univariate analysis was less significant and the test accuracy was lower. Figure 4 displays high-risk and low-risk distribution for each multilocus genotype combination of the identified tetragenic model. The interaction patterns of high risk and low risk cells varied across each of the different multilocus dimensions, which showed evidence of epistasis, or gene-gene interaction.

**Table 3:** Interaction SNPs detected among *CHRNB2*, *NTRK2*, *BDNF*, and *CHRNA4*.

Model^a^	TA^b^	*p*_sign_ value^c^	*p*_perm_ value^d^
rs2072660-rs1209068-rs11030134-rs6011770			
GEE-GMDR	.5780	<10e-04	2.62e-04
GMDR_ FTND_ e	.5411	6.30e-02	9.87e-02
GMDR _ND_ f	.5283	2.41e-02	2.40e-01
GMDR_ MC_ g	.5128	5.40e-01	7.86e-01

a In the model, from left to right, the SNPs are located in *CHRNB2*, *NTRK2*, *BDNF*, *CHRNA4*, respectively.

b TA denotes test accuracy.

c *p*_sign_ values were from the sign test after Bonferroni correction.

d *p*_perm_ values were from the permutation test after Bonferroni correction.

e FTND denotes the Fagerstrom Test for Nicotine Dependence.

f ND denotes the DSM4 Nicotine Dependence.

g MC denotes the Maximum number of Cigarette.

**Table 4:** Information on the SNPs in the best model identified using GEE-GMDR method.

SNP	Gene	Chromosome	Domain	Physical Position	Alleles^a^	Reported MAF^b^
rs2072660	*CHRNB2*	1	3UTR	152815345	C/**T**	.319
rs1209068	*NTRK2*	9	Intron	86530338	C/**T**	.096
rs11030134	*BDNF*	11	5′ Flanking	27743050	**C**/T	.282
rs6011770	*CHRNA4*	20	Intron	61447875	C/**T**	.054

a The nucleotide of each SNP shown in bold represents the minor allele as given in dbSNP (build 138).

b The minor allele frequency (MAF) presented in dbSNP (build 138).

Recent evidence revealed genetic associations between ND and *CHRNA4* [41], [42], *NTRK2* and *BDNF* [43]. Biochemical studies have showed that the α4β2-containing nAChR subtype make up the majority of the high-affinity nicotine-binding sites in our brain and that the expression of genes of both subunits are increased in the case of chronic nicotine exposure. The binding of *NTRK2* to *BDNF* regulates the short-term synaptic functions and long-term potentiation of brain synapses. Biological interactions of *BDNF* with *NTRK2* and *CHRNA4* with *CHRNB2* have been constructed experimentally under vitro and animal models. Statistical interactions among *CHRNA4*, *CHRNB2*, *BDNF* and *NTRK2* underlying ND had been discovered in our previous studies [25], [44], [45]. Our research results using novel multivariate method indicate that there are potential interactions among these four genes. The finding requires further investigation both through in silico analysis and laboratory verification in future.

## Discussion

Identification of epistatic and/or gene-environment interactions underlying complex traits variation is one of the important and challenging tasks in human genetics and epidemiology studies. In the literature, joint actions of multiple factors are the norm rather than the exception in the genetic architecture of complex traits [13]–[16]. Compared with traditional methods established by an extension of single-factor-based statistical linear model, recently emerging combinatorial approaches such as MDR, the CPM, the RPM and the GMDR could bridge between statistical interaction and biological mechanism, and take biological plausibility into account. Relative to a wealth of univariate methods available, however, the statistical methods for detecting interactions underlying multiple traits are less well developed for these combinatorial approaches.

Complex diseases can be multidimensional and defined by a set of intermediate phenotypes that cluster together and are correlated. Complex traits can often be grouped into symptom groups. In some cases, data are collected from longitudinal studies. Using a univariate analysis, instead of multivariate analysis, will substantially reduce the power or likely yield a misleading result. It is desirable to to extend GMDR method for multivariate data to analyze complex multifaceted disorders and longitudinal data and/or toincrease test power. Here, we have proposed a natural extension of GMDR to multiple phenotypes based on GLM, QLM and GEE models in terms of the nature of data available, named multivariate GMDR approaches. In GLM, we need to specify all the probability distribution, the relationship between the mean and the variance, and the covariance. In QLM, we only need to specify the relationship between the mean and the variance, and the multivariate correlation structure. GEE model requires only the relationship between the mean and the variance of the marginal distribution. GEE, the extension of GLM and QLM, is more flexible, and has been widely applied to correlated multivariate data because of its robustness and no need for specifying the exact covariance structure [29], [30], [46]. GEE can yield consistent and efficient estimation of coefficient parameters for the multivariate data, even though the assumed working correlation structure is incorrect and the data are missing completely at random [29]. The GEE-GMDR can therefore handle a variety of multivariate data with different correlation structures, for example, repeated data, longitudinal data, cluster data and multiple phenotypes, under the assumption of estimation of arbitrary subset of the covariance parameters [46]. In conclusion, we propose a comprehensive statistical framework for detection of interactions underlying multivariate phenotypes. Within this framework, various kinds of phenotypes with diverse correlation structures, such as categorical data and continuous data can be analyzed. The original GMDR can be regard as a special case of multivariate GMDR, when the cluster size of multivariate data is equal to one.

One of the key advantages of the proposed method, as shown in both the simulation and the real data analyses of this study, is that this new multivariate approach is more powerful via borrowing information across multiple correlated phenotypes. We have compared the power of GEE-GMDR with the univariate GMDR method through Monte Carol simulations under the digenic, trigenic and tetragenic interaction models. Remarkable increase in power has been observed using our proposed multivariate method in the simulations, especially for trigenic and tetragenic models. The lower power of identifying high order interaction may mostly be attributed to smaller sample size, low allele frequency and low genetic variance [26]. Here, the multivariate method can be an appropriate and promising approach to search for weak associations of high order combination of multiple factors underlying complex traits, which may be missing when each phenotype is considered independently. Consistent with other multivariate techniques, we also observed that the power of GEE-GMDR depends on the size and the structure of residual correlation between phenotypes [6], [37]–[39]. The power to identify a pleiotropic gene-gene interaction is greatly improved in a case with a negative residual correlation. Such a pattern is observed in different interaction models.

The proposed approach has been applied to the SAGE sample with inclusion of multiple phenotypes related to ND. Conventional single-marker methods that separate interacting genes from their context fail to interpret the whole genetic architecture and seem to be inefficient. It is appropriate and powerful to identify high order gene-gene interaction underlying the ND, which is controlled by a large number of genes with a modest effect size, as demonstrated in our study and previous reports [25], [44], [45]. In human genetic studies, we often collect many correlated phenotypes. Correction for multiple testing is also required in individual analyses for multiple phenotypes. However, the proposed method analyzes all of the traits simultaneously and jointly in a unified statistical model, offering a protected overall significance level.

Very recently a multivariate GMDR method based on GEE was just proposed in the literature [32]. However, our statistic is more general and powerful and that method [32] can be viewed as a special case of our approaches. For data from repeated measurement study, our method is identical to that approach, but will be different for longitudinal data, cluster data and multiple phenotypes. Given the difference of joint genetic effect of genes on multiple phenotypes, the proposed score statistic is more appropriate to model relationships between phenotypes. In addition, our proposed method is evaluated through simulation studies and the real data analysis for quantitative traits; whereas the method in Chio and Park [32] is only assessed by the real data studies for few binary traits. The performance of high-order interactions and the influence of residual correlation on power using GEE-GMDR method are also investigated in our study. Further, we also corrected for population stratification in the real data analysis. In this way our proposed method is a unified GEE-GMDR that can handle a variety of data.

As is the same as the original GMDR, computational limitations for high dimensional interactions still remain with our proposed method, especially for identifying high order interaction for data of the genome wide association studies (GWAS). However, Zhu *et al* have developed a graphics processing unit (GPU)-based GMDR program (GMDR-GPU) [47], which can handle GWAS data and run more faster than the original GMDR software. Through combination of our GEE-GMDR algorithm with GMDR-GPU program, the problem of computational expense can be alleviated.

GEE statistic used in our GEE-GMDR approach makes use of all dimensions of data but sometimes a part of them will dilute useful information. The possible directions for future research are to explore constructing an overall or extracting few summary statistics by multivariate statistical techniques (e.g., principal components analysis and canonical correlation analysis). Principal components may be useful, in particular, when the phenotypes of interest are highly correlated and/or highly dimensional, i.e., nearly collinear or degenerated multivariate data. The principle is to construct the test statistic based on the first few principal components from a principal components analysis or canonical correlation analysis, instead of all components. Principal components analysis or canonical correlations, as a kind of statistical method of linear transformations, are effective to reduce the number of phenotypes surveyed.
